# Trimethylamine N-Oxide Does Not Impact Viability, ROS Production, and Mitochondrial Membrane Potential of Adult Rat Cardiomyocytes

**DOI:** 10.3390/ijms20123045

**Published:** 2019-06-21

**Authors:** Giulia Querio, Susanna Antoniotti, Renzo Levi, Maria Pia Gallo

**Affiliations:** Department of Life Sciences and Systems Biology, University of Turin, Via Accademia Albertina 13, 10123 Turin, Italy; giulia.querio@unito.it (G.Q.); susanna.antoniotti@unito.it (S.A.); renzo.levi@unito.it (R.L.)

**Keywords:** trimethylamine N-oxide, cardiomyocytes, cardiotoxicity, ROS, mitochondrial membrane potential

## Abstract

Trimethylamine N-oxide (TMAO) is an organic compound derived from dietary choline and L-carnitine. It behaves as an osmolyte, a protein stabilizer, and an electron acceptor, showing different biological functions in different animals. Recent works point out that, in humans, high circulating levels of TMAO are related to the progression of atherosclerosis and other cardiovascular diseases. However, studies on a direct role of TMAO in cardiomyocyte parameters are still limited. The purpose of this work is to study the effects of TMAO on isolated adult rat cardiomyocytes. TMAO in both 100 µM and 10 mM concentrations, from 1 to 24 h of treatment, does not affect cell viability, sarcomere length, intracellular ROS, and mitochondrial membrane potential. Furthermore, the simultaneous treatment with TMAO and known cardiac insults, such as H_2_O_2_ or doxorubicin, does not affect the treatment’s effect. In conclusion, TMAO cannot be considered a direct cause or an exacerbating risk factor of cardiac damage at the cellular level in acute conditions.

## 1. Introduction

Trimethylamine N-oxide (TMAO) is an amine oxide directly introduced through diet or synthetized from its precursors, primarily L-carnitine and choline, that are transformed into trimethylamine (TMA) by the gut microbiota. Once absorbed, TMA in most mammals is oxidized by hepatic FMOs to form TMAO which enters the systemic circulation. Several studies illustrate different biological functions of TMAO in other animals. In elasmobranchs and deep-sea fishes, it acts as an osmolyte able to counteract either osmotic or hydrostatic pressure. It is a protein stabilizer preserving protein folding and it also acts as an electron acceptor balancing oxidative stress [1,2]. TMAO is also reduced to TMA in the anaerobic metabolism of a number of bacteria. Although TMAO is involved in several reactions within cells, recent studies highlight its detrimental role when present in high plasmatic concentrations in some mammals. In fact, TMAO seems to be involved in accelerating endothelial cell senescence, enhancing vascular inflammation and oxidative stress [3,4]; it also could be involved in the stimulation of platelet hyperreactivity and in the onset of thrombosis, exacerbating atherosclerotic lesions [5]. Several studies also underline the role of TMAO in the pathogenesis of type 2 diabetes mellitus [6]. There are limited data on its function in mediating direct cardiac injuries, and those studies are mainly focused on its role in the impairment of mitochondrial metabolism [7] and calcium handling [8]. On the contrary, recent papers are re-evaluating the role of TMAO, underlining the emerging debate on its direct effect in causing or exacerbating cardiovascular diseases (CVD) [9,10]. First criticisms point out that populations having diets with high concentrations of TMAO, like those rich in fish products, when compared to Western diets rich in its precursors, have reduced risks of CVD or diseases assumed to be related to high TMAO plasma levels [11]. Another study demonstrates that TMAO does not affect macrophage foam cell formation and lesion progression in ApoE^−/−^ mice expressing human cholesteryl ester transfer protein, suggesting that the molecule does not worsen atherosclerosis [12]. Furthermore, administration of TMAO seems to improve symptoms related to streptozotocin-induced diabetes in rats and mice, highlighting no direct contribution of the molecule in exacerbating this condition [13]. Finally, data about TMAO plasma concentrations in healthy and pathological subjects are not clear: the lack of plasma concentration ranges of the molecule highlights the difficulties in referring to TMAO as a protective or a damaging factor in CVD. Starting from these conflicting considerations, the aim of this work was to evaluate for the first time the effect of TMAO in an in vitro model of adult rat cardiomyocytes exposed to different concentrations of the compound from 1 h to 24 h of treatment. To show whether TMAO exacerbates or reduces induced cell stress, cardiomyocytes were simultaneously treated with TMAO and H_2_O_2_ or doxorubicin (DOX). Investigations were focused on cell viability after TMAO or TMAO and stressors co-treatment, assessing cell morphology and functionality with α-actinin staining and specific probes that measure oxidative stress status and mitochondrial membrane potential.

## 2. Results

### 2.1. TMAO and Cell Viability

**Figure 1:**
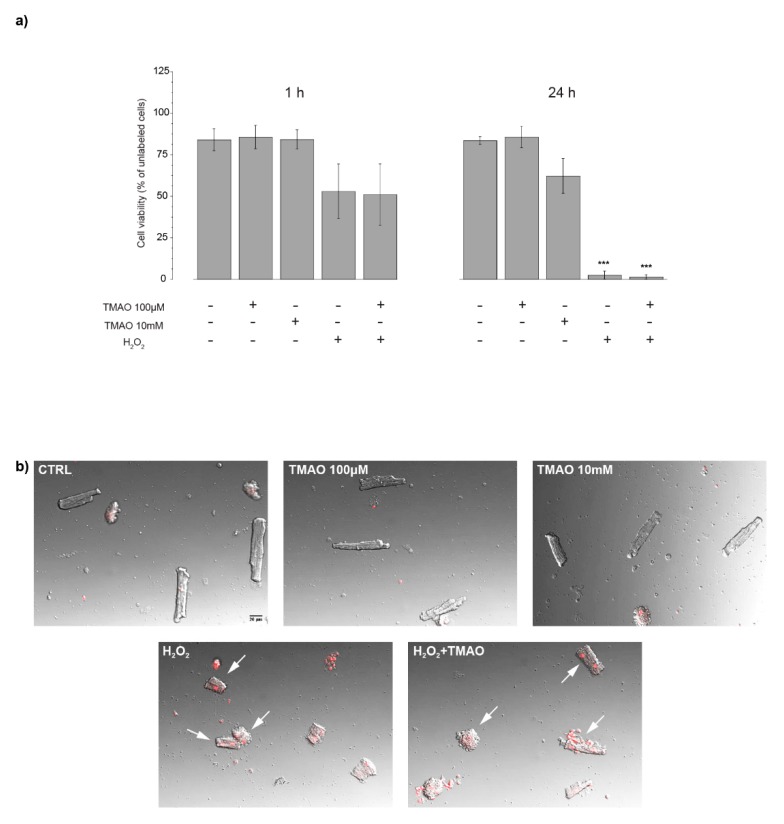
Cell viability after trimethylamine N-oxide (TMAO) exposure. (**a**) Bar graph of cell viability after 1 h and 24 h of treatment. Cell viability results reduced only after H_2_O_2_ treatment for 24 h, condition not improved or worsened by the simultaneous treatment with TMAO (refer to the main text for numerical values). (**b**) Merged images in bright field and fluorescence of cells treated for 24 h with TMAO 100 µM and TMAO 10 mM, H_2_O_2_, and H_2_O_2_+TMAO and labeled with propidium iodide (PI) (20× magnification). White arrows point out PI-stained, damaged cardiomyocytes.

In order to investigate the effect of TMAO on cell viability, cardiomyocytes were treated with TMAO 100 μM, TMAO 10 mM, H_2_O_2_ 50 μM, and H_2_O_2_ 50 μM + TMAO 100 μM. After 1 h or 24 h of treatment, cardiomyocytes were labeled with propidium iodide (PI) and marked nuclei of suffering cells were detected by confocal microscopy at 568 nm. Concentrations used were taken from the literature: TMAO 100 µM is recognized as a marker of cardiovascular risk, TMAO 10 mM is over the physiological range and was tested here to detect any effect induced by high concentrations of the compound [14]. As shown in Figure 1a, there is no effect of TMAO 100 µM or TMAO 10 mM at either time of treatment, whereas H_2_O_2_, used here as a positive control, had effects only after 24 h. Simultaneous treatment with H_2_O_2_ and TMAO did not improve or worsen the effect of the stressor on cell viability (1 h—CTRL: 83.95 ± 6.59, *n* = 3, 52 cells; TMAO 100 µM: 85.52 ± 7.01, *n* = 5, 81 cells; TMAO 10 mM: 84.08 ± 5.84, *n* = 5, 92 cells; H_2_O_2_: 52.92 ± 16.46, *n* = 3, 56 cells; H_2_O_2_ + TMAO: 50.93 ± 18.50, *n* = 3, 58 cells. 24 h—CTRL: 83.42 ± 2.29, *n* = 3, 101 cells; TMAO 100 µM: 85.66 ± 6.48, *n* = 3, 91 cells; TMAO 10 mM: 62.22 ± 10.47, *n* = 3, 119 cells; H_2_O_2_: 2.38 ± 2.38, *n* = 3, 82 cells (*** *p* < 0.001); H_2_O_2_ + TMAO: 1.33 ± 1.33, *n* = 3, 41 cells (*** *p* < 0.001)). Figure 1b displays confocal images of cardiomyocytes from a representative experiment of PI staining after 24 h of treatment. White arrows point out PI-stained, damaged cardiomyocytes.

### 2.2. TMAO and Sarcomere Length

**Figure 2:**
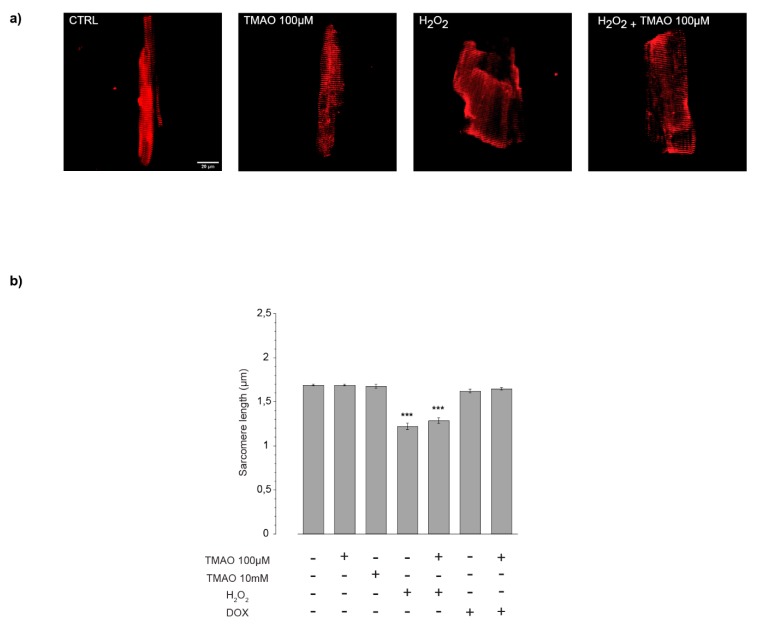
Sarcomere length after TMAO treatment. (**a**) Confocal microscopy images of fixed cells labeled for α-actinin protein (40× magnification). (**b**) Bar graph showing sarcomere length after 24 h of treatment with TMAO and other stressors: no cell shrinkage is measured when cells are exposed to different TMAO concentrations (refer to the main text for numerical values).

To evaluate if TMAO was able to alter sarcomere structures after 24 h of treatment, sarcomere length was measured in α-actinin-stained cardiomyocytes. As shown in Figure 2, no changes in sarcomere length were observed in cells treated with TMAO, while H_2_O_2_ 50 μM used as a positive control caused cardiomyocyte shrinkage, a condition that was not improved or worsened by the simultaneous treatment with TMAO. In cardiomyocytes treated with DOX 1 µM for 24 h, no sarcomere length variations were observed, because the DOX treatment in our model was designed to induce mild damage preceding cell shortening. Even so, TMAO 100 µM did not modify DOX-treated cardiomyocytes (sarcomere length in µm—CTRL: 1.69 ± 0.01, *n* = 7, 42 cells; TMAO 100 µM: 1.69 ± 0.01, *n* = 6, 34 cells; TMAO 10 mM: 1.67 ± 0.02, *n* = 3, 19 cells; H_2_O_2_: 1.22 ± 0.04, *n* = 5, 33 cells (*** *p* < 0.001); H_2_O_2_ + TMAO: 1.28 ± 0.03, *n* = 3, 24 cells (*** *p* < 0.001); DOX: 1.62 ± 0.02, *n* = 3, 16 cells; DOX + TMAO: 1.65 ± 0.01, *n* = 3, 15 cells).

### 2.3. TMAO and Intracellular Reactive Oxygen Species (ROS)

**Figure 3:**
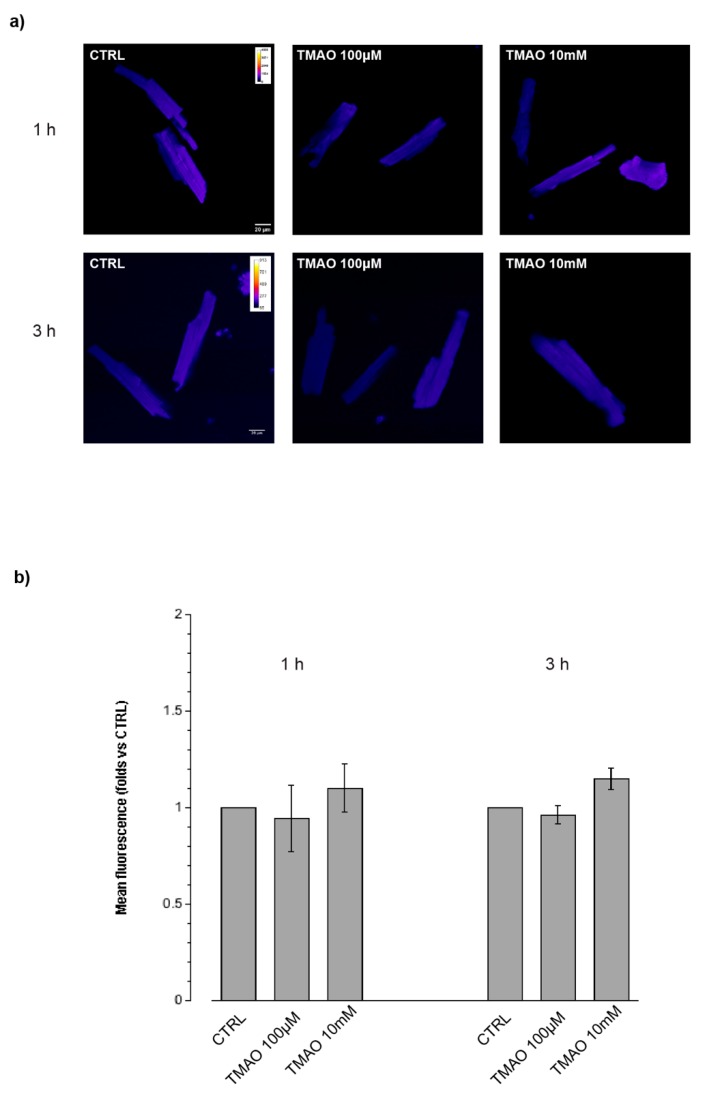
ROS production after 1 h and 3 h of treatment. (**a**) Confocal microscopy images of cells treated with TMAO 100 µM and TMAO 10 mM for 1 h and 3 h and labeled with the DCF-DA probe (60× magnification). (**b**) Bar graph showing mean fluorescence after 1 h and 3 h of treatment, no variations or ROS produced are detectable (refer to the main text for numerical values).

**Figure 4:**
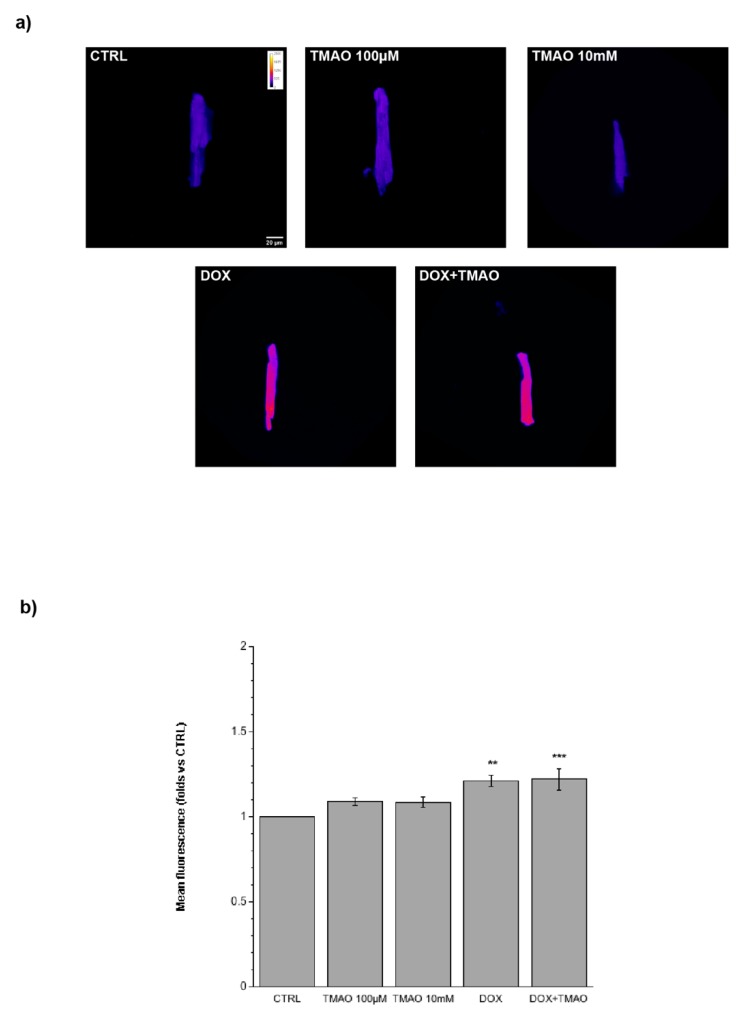
ROS production after 24 h of treatment. (**a**) Confocal microscopy images of cells treated with TMAO 100 µM and TMAO 10 mM for 24 h and labeled with the DCF-DA probe. In these experiments, doxorubicin (DOX) is used as a positive control (60× magnification). (**b**) Bar graph showing mean fluorescence after 24 h of treatment. No variations of ROS produced are detectable after TMAO treatment, and a small but significant increase is visible after DOX treatment (used here as a positive control); this increase is not changed by a simultaneous treatment with TMAO (refer to the main text for numerical values).

In order to determine a variation in total ROS produced after treatment with TMAO for 1 h, 3 h, or 24 h, cells were labeled with the DCF-DA probe and its fluorescence was quantified and related to the control. As shown in Figure 3 (1 h, 3 h) and 4 (24 h), no fluorescence variations after TMAO treatment were detected at any concentration and time used. As a positive control, we employed DOX 1 µM for 24 h [15]; this drug caused a significant variation in ROS production with respect to the control condition. TMAO 100 µM did not modify ROS production in the DOX-treated cardiomyocytes (Figure 4) (1 h—TMAO 100 µM: 1.30 ± 0.21, *n* = 3, 34 cells; TMAO 10 mM: 1.32 ± 0.23, *n* = 3, 40 cells, vs. CTRL; 3 h—TMAO 100 µM: 0.96 ± 0.05, *n* = 3, 45 cells; TMAO 10 mM: 1.15 ± 0.09, *n* = 3, 52 cells, vs. CTRL; 24 h—TMAO 100 µM: 1.18 ± 0.04, *n* = 5, 40 cells; TMAO 10 mM: 1.24 ± 0.16, *n* = 3, 52 cells; DOX: 1.33 ± 0.11, *n* = 4, 21 cells (** *p* < 0.01); DOX+TMAO: 1.27 ± 0.01, *n* = 6, 31 cells (*** *p* < 0.001), vs. CTRL).

### 2.4. TMAO and Mitochondrial Membrane Potential

**Figure 5:**
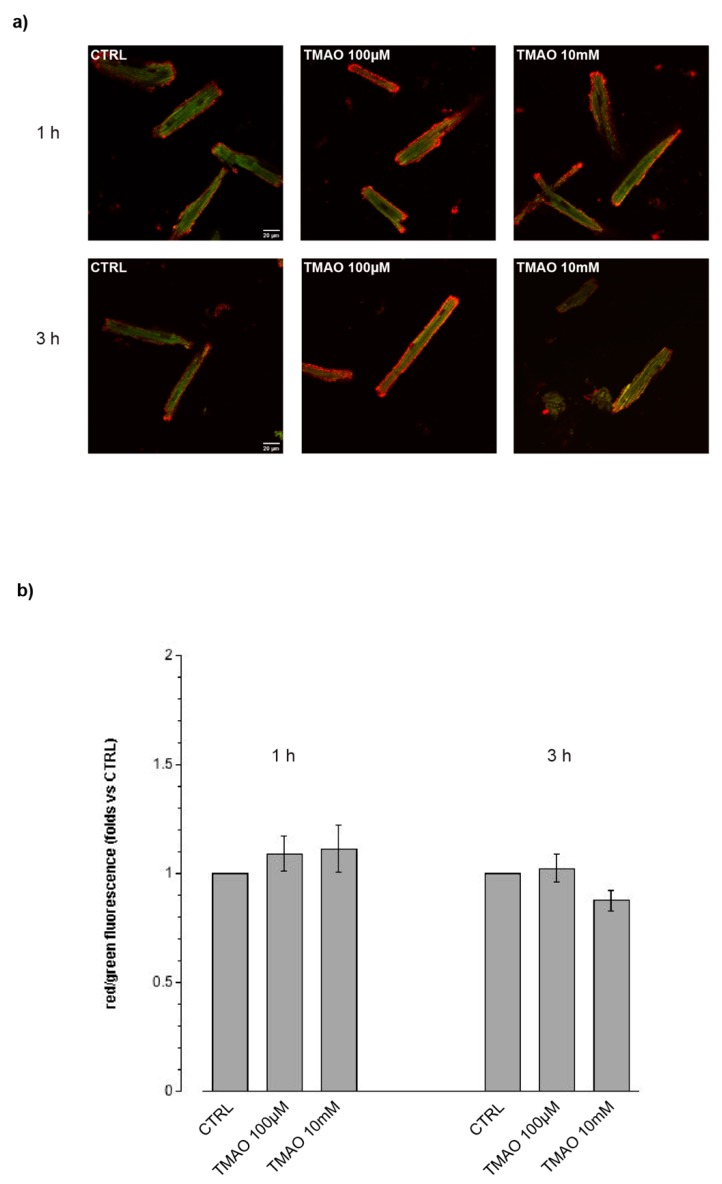
Mitochondrial membrane potential variation following 1 h and 3 h of treatment. (**a**) Confocal microscopy images of cells treated with TMAO 100 µM and TMAO 10 mM for 1 h and 3 h and labeled with the JC-1 probe (60× magnification). (**b**) Bar graph showing red/green fluorescence after 1 h and 3 h of treatment, no variations of mitochondrial membrane potential are detected (refer to the main text for numerical values).

**Figure 6:**
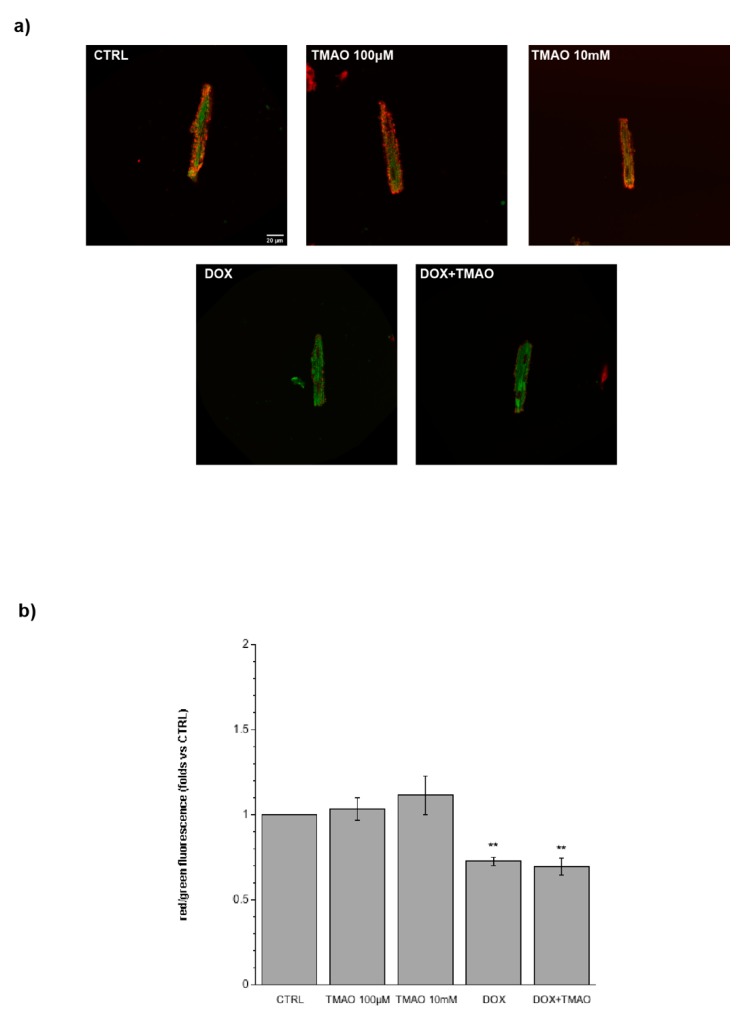
Mitochondrial membrane potential variation following 24 h treatment. (**a**) Confocal microscopy images of cells treated with TMAO 100 µM and TMAO 10 mM for 24 h and labeled with the JC-1 probe. In these experiments, doxorubicin (DOX) is used as a positive control (60× magnification). (**b**) Bar graph showing red/green fluorescence after 24 h of treatment, no variations of mitochondrial membrane potential are detected, a little but significant reduction of the ratio is visible after DOX treatment used here as a positive control, condition not changed in a simultaneous treatment with TMAO (refer to the main text for numerical values).

To investigate the potential metabolic damage induced by TMAO, cardiomyocytes treated with TMAO 100 µM and 10 mM for 1 h, 3 h, or 24 h were labeled with the JC-1 probe. Figure 5 (1 h, 3 h) and 6 (24 h) show variations in mitochondrial membrane potential (red/green fluorescence ratio) detected by confocal microscopy in living cells. TMAO treatment from 1 to 24 h did not cause any difference with respect to the control, indicating no mitochondrial effect of the molecule, whereas, as expected, DOX caused a depolarization of mitochondrial membrane potential after 24 h of treatment. TMAO 100 µM did not modify mitochondrial membrane potential in DOX-treated cardiomyocytes (Figure 6) (1 h—TMAO 100 µM: 1.09 ± 0.08, *n* = 5, 65 cells; TMAO 10 mM: 1.11 ± 0.11, *n* = 3, 49 cells, vs. CTRL. 3 h—TMAO 100 µM: 1.02 ± 0.06, *n* = 3, 26 cells; TMAO 10 mM: 0.88 ± 0.05, *n* = 4, 39 cells, vs. CTRL; 24 h—TMAO 100 µM: 1.08 ± 0.11, *n* = 3, 21 cells; TMAO 10 mM: 0.95 ± 0.15, *n* = 3, 54 cells; DOX: 0.73 ± 0.02, *n* = 3, 24 cells (** *p* < 0.01); DOX + TMAO: 0.69 ± 0.05, *n* = 3, 22 cells (** *p* < 0.01), vs. CTRL).

## 3. Discussion

This study provides novel insights into the physiological role of TMAO in isolated adult rat cardiomyocytes. Our findings do not show effects of TMAO on cell viability, sarcomere length, ROS production, and mitochondrial membrane potential within the range of concentration used. Moreover, we demonstrate that TMAO does not exacerbate or counteract the effect of known insults, such as H_2_O_2_ or doxorubicin, tested for up to 24 h of treatment. Taken together, these results suggest that TMAO should not be considered a primary cause of acute cardiac damage and that the molecule could not revert or worsen existing risk factors of cardiac damage.

In the last few years, many studies suggest a strong relationship between diet, gut microbiota, and cardiovascular diseases [16]. In particular, some attention has been pointed to either TMAO directly coming from diet (fish), or produced from L-carnitine and choline conversion by gut microbiota into TMA and oxidized in the liver by FMO3 enzymes [14,17].

Experiments have mainly now focused on the role of TMAO in damaging endothelial cells. It has been described as upregulating cellular senescence, thereby reducing cell proliferation, increasing the expression of senescence markers, such as p53 and p21, and impairing cell migration [3]. TMAO also increases the oxidative stress of endothelial cells through a down-regulation of SIRT-1 and impairs NO production that causes endothelial dysfunction [4]. Hypertensive effects of TMAO have been evaluated by Ufnal and colleagues who demonstrated that TMAO has a role in stabilizing the action of Ang II and in prolonging its hypertensive effect, underlining the role of TMAO in stabilizing protein conformation and no direct role of the molecule in mediating hypertension [18]. Koeth and colleagues underlined the strong relationship between the high consumption of TMAO precursors, high TMAO plasma concentrations, and the development of atherosclerosis [19], while another study underlined the effect of the metabolite in enhancing platelet hyperreactivity and thrombosis risk in subjects with high TMAO plasma concentrations [5]. With respect to the cardiovascular effects of TMAO, Dambrova and collaborators showed that high plasma concentrations of the molecule are linked with increased body weight and insulin resistance and that it directly correlates with an augmented risk of diabetes [20].

Only a few studies are centered on the direct effect of the molecule on cardiac cells; in particular, they focus on the impairment of mitochondrial metabolism in the heart and underline TMAO as an agent that increases the severity of cardiovascular events or that enhances the progression of cardiovascular diseases [7]. Savi and colleagues showed a damaging effect of TMAO in cardiomyocytes because it worsens intracellular calcium handling with a reduced efficiency in the intracellular calcium removal and consequent loss in functionality of cardiac cells; furthermore, TMAO seems to alter energetic metabolism and to facilitate protein oxidative damage [8].

This scenario presents TMAO as either a marker or a direct agent involved in vascular and cardiac outcomes, but recent papers seem to oppose this point of view, highlighting uncertainty about the causative relation between TMAO and CVD [9]. The function of TMAO is still being debated, for example, the controversy surrounding fish-rich diets, because of the higher bioavailability of the compound in seafood products and their well-known role in lowering risk of CVD. Additionally, TMAO does not enhance atherosclerosis development because it seems not to be involved in foam cell formation even at higher concentrations than physiological ones [12], and there is no direct correlation between high plasma TMAO concentrations and coronary heart diseases [21,22]. Finally, findings by Huc et al. have also underlined a protective role of TMAO in reducing diastolic dysfunction and fibrosis in the pressure-overloaded heart [23].

The present study fits into this debate and the results presented agree with other works supporting TMAO as a non-damaging factor. In fact, it is well known that the loss of vital cardiac cells is a damaging condition that hampers primarily the functionality of the heart and has several aggravating responses also in peripheral tissues. Our first investigations underline no toxic effect in cardiomyocytes exposed to high concentrations of TMAO, highlighting the result that the molecule is not involved in inducing cardiac tissue cell loss, and no alterations of cardiac structure emerge from the evaluation of sarcomere length and cytoskeletal organization. Oxidative stress could be considered one of the causative factors of senescence in cells and one of the promoters of cardiometabolic reorganization in response to injury. With respect to TMAO as a possible inducer of ROS rising, both in a cytoplasmic and a mitochondrial environment, we show no variation in ROS production even after 24 h of treatment and we detect no depolarization of mitochondrial membrane potential, underlining the result of no direct influence by the molecule in inducing cardiac cell senescence.

## 4. Materials and Methods

### 4.1. Animal Care and Sacrifice

Experiments were performed on female adult rats which were allowed ad libitum access to tap water and standard rodent diet. The animals received human care in compliance with the Guide for the Care and Use of Laboratory Animals published by the US National Institutes of Health (NIH Publication No. 85-23, revised 1996), and in accordance with Italian law (DL-116, Jan. 27, 1992). The scientific project was supervised and approved by the Italian Ministry of Health, Rome, and by the ethical committee of the University of Torino (approval code 116/2017-PR, 3/2/2017). Rats were anaesthetized by i.p. injection of tiletamine (Zoletil 100, Virbac, Carros, France) and sacrificed by stunning and cervical dislocation.

### 4.2. Solutions and Drugs

Tyrode standard solution containing (in mM): 154 NaCl, 4 KCl, 1 MgCl_2_, 5.5 D-glucose, 5 HEPES, 2 CaCl_2_, pH adjusted to 7.34 with NaOH. Ca^2+^ free Tyrode solution containing (in mM): 154 NaCl, 4 KCl, 1 MgCl_2_, 5.5 D-glucose, 5 HEPES, 10 2,3-Butanedione monoxime, 5 taurine, pH 7.34. All drug-containing solutions were prepared fresh before the experiments and the Tyrode solutions were oxygenated (O_2_ 100%) before each experiment. Unless otherwise specified, all reagents for cell isolation and experiments were purchased from Sigma-Aldrich (St. Louis, MO, USA).

### 4.3. Adult Rat Ventricular Cell Isolation

Isolated cardiomyocytes were obtained from the hearts of adult rats (200–300 g body weight) according to the previously described method [24]. Briefly, after sacrifice, rat heart was explanted, washed in Ca^2+^ free Tyrode solution, and cannulated via the aorta. All the following operations were carried on under a laminar flow hood. The heart was perfused at a constant flow rate of 10 mL/min with Ca^2+^ free Tyrode solution (37 °C) with a peristaltic pump for approximately 5 min to wash away the blood and then with 10 mL of Ca^2+^ free Tyrode supplemented with collagenase (0.3 mg/mL) and protease (0.02 mg/mL). Hearts were then perfused and enzymatically dissociated with 20 mL of Ca^2+^ free Tyrode containing 50 µM CaCl_2_ and the same enzymatic concentration as before. Atria and ventricles were then separated and the ventricles were cut in small pieces and shaken for 10 min in 20 mL of Ca^2+^ free Tyrode solution in the presence of 50 µM CaCl_2_, collagenase, and protease. Calcium ion concentration was slowly increased to 0.8 mM. Cardiomyocytes were then plated on glass cover slips or glass bottom dishes (Ibidi, Martinsried, Germany), both treated with laminin to allow cell adhesion.

### 4.4. Cell Viability

Cell viability was evaluated by propidium iodide (PI) staining on glass bottom dishes for adherent cells. At the end of the treatments, cells were incubated with PI (10 µg/mL, Invitrogen, Carlsbad, CA, USA) for 5 min in the dark. Nuclei of suffering cells were detected with confocal microscopy using an Olympus Fluoview 200 microscope (Shinjuku, Tokyo, Japan) at 568 nm (magnification 20×). Merged images were created with ImageJ (U.S. National Institutes of Health, Bethesda, MD, USA, https://imagej.nih.gov/ij/) and cell viability was calculated as percentage of (total cells-labeled cells)/total cells.

### 4.5. Evaluation of Sarcomere Length

Cardiomyocytes on glass coverslips were stimulated with TMAO and with H_2_O_2_ as a positive control. Cells were treated for 24 h with TMAO, at 100 µM and 10 mM, then the sarcomere protein α-actinin, localized in the Z lines, was detected using confocal microscopy. Subsequently, cells were fixed in 4% PFA for 40 min. After two washes with PBS, cells were incubated for 20 min with 0.3% Triton and 1% bovine serum albumin (BSA) in PBS and stained for 24 h at +4 °C with a mouse monoclonal anti-α-actinin primary antibody (Sigma-Aldrich, 1:800). Cover slides were washed twice with PBS and incubated 1 h at room temperature with the secondary antibody (1:2000, anti-mouse Alexa Fluor 568, Thermo Fisher Scientific, Waltham, MA, USA). After two washes in PBS, coverslips were mounted on standard slides with DABCO and observed after 24 h under a confocal microscope. Confocal fluorimetric measurements were acquired using a Leica SP2 laser scanning confocal system (Wetzlar, Germany), equipped with a 40× water-immersion objective. Image processing and analysis were performed with ImageJ software. Sarcomere length was evaluated measuring the distance between Z lanes in *n* = 10 sarcomeres/cell.

### 4.6. Intracellular Reactive Oxygen Species (ROS) Measurement

Production of ROS was evaluated by fluorescence microscopy using a 2′-7′-dichlorofluorescein diacetate probe (DCF-DA). After adhesion on glass bottom dishes, DCF-DA solution (5 µg/mL) was added to each dish 30 min prior to the end of the treatment, then the cells were washed with standard Tyrode solution. Fluorescence images at 488 nm were acquired using an Olympus Fluoview 200 microscope (magnification 60×). Fluorescence variations were calculated with the definition and measurement of regions of interest (ROIs) using ImageJ software and expressed as relative Medium Fluorescence Index (MFI) compared to control, fixed at 1.

### 4.7. Mitochondrial Membrane Potential Measurement

Mitochondrial membrane potential was evaluated by staining cardiomyocytes with the dye 5,5′,6,6′-tetrachloro-1,1′,3,3′-tetraethyl-imidacarbocyanine iodide (JC-1). JC-1 solution (10 µM) was added to each dish 30 min prior to the end of the treatment, then the cells were washed with standard Tyrode solution. Fluorescence images at 488 nm and 568 nm were acquired using an Olympus Fluoview 200 microscope (magnification 60×). Amounts of the monomeric form of the dye were quantified using the red/green fluorescence ratio in the ROIs using ImageJ software and expressed as folds towards control, fixed at 1.

### 4.8. Statistical Analysis

All data are expressed as mean ± standard error of the mean. For differences between mean values, Bonferroni’s multiple comparisons test was performed. Differences with *p* < 0.05 were regarded as statistically significant.

## 5. Conclusions

**Figure 7:**
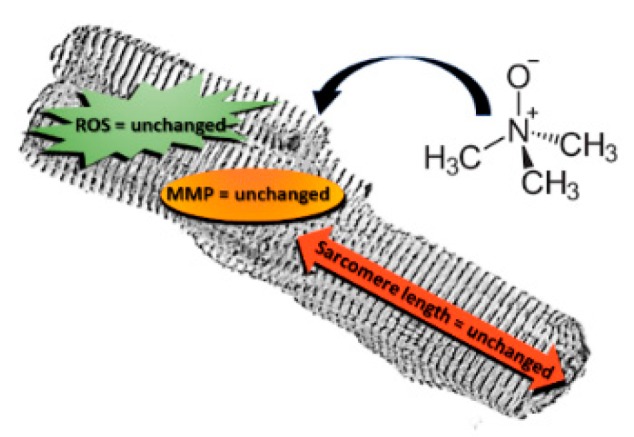
No effects of TMAO are detectable on isolated adult rat cardiomyocyte viability, sarcomere length, ROS production, and mitochondrial membrane potential.

In summary, this study demonstrates that TMAO is not directly involved in causing or exacerbating cardiac damage in an acute stress model (Figure 7). However, this study has some limitations: a very wide range of plasmatic TMAO concentrations have been presented in literature, within different mammals, and also between different sexes of the same species; so, several orders of magnitude can be considered physiological [25,26]. Therefore, to test the direct effect of the molecule, high concentrations were used, even higher than human physiological ones. Another weakness of the study could be linked to the time of treatment, because the evaluations were no longer than 24 h and they only represented an acute exposure to TMAO. Moreover, in order to better evaluate the mechanism involved in TMAO-mediated responses, it may be necessary to treat cells for a longer period to assess a chronic stress compatible with the development of CVD. Furthermore, we only studied the TMAO effect on female ventricular cardiomyocytes and it may be interesting to extend the analysis also to male cardiomyocytes as gender differences have been observed in cardioprotective mechanisms [27] and TMAO-induced intracellular calcium imbalance has been described in male cardiomyocytes [8]. Finally, our findings provide new insights into the cardiac effect of TMAO, exploring the direct treatment of isolated cardiomyocytes.

## Abbreviations

TMAO	Trimethylamine N-oxide
PI	Propidium iodide
ROS	Reactive oxygen species
DCF-DA	2′-7′-Dichlorofluorescein diacetate
JC-1	5,5′,6,6′-Tetrachloro-1,1′,3,3′-tetraethyl-imidacarbocyanine iodide
